# The metabolomic profile of psoriatic arthritis patients unveils the unbalance of disease-related molecules and pathways

**DOI:** 10.1038/s41598-025-34101-4

**Published:** 2025-12-30

**Authors:** M. M. Angioni, C. Piras, V. P. Leoni, A. Floris, M. Spada, K. Lilliu, M. Congia, E. Chessa, M. Piga, L. Atzori, A. Cauli

**Affiliations:** 1 Rheumatology Unit, Department of Medical Sciences and Public Health, AOU and University of Cagliari, Monserrato, Cagliari, Italy; 2 Clinical Metabolomics Unit, Department of Biomedical Sciences, University of Cagliari, Monserrato, Cagliari, Italy

**Keywords:** Psoriatic arthritis, Metabolomics, Pathogenesis, Disease activity, DAPSA, Diabetes, Metabolic syndrome, Glucose, BCCA, Biochemistry, Biomarkers, Diseases

## Abstract

Among the unmet needs in psoriatic arthritis (PsA), the precise mechanisms underlying the contribution of genetic susceptibility, environmental triggers and systemic inflammation are still under investigation. As a complex and multi-factorial condition, metabolomics in PsA may offer additional insight into pathways driving the pathogenesis, allowing for a better understanding of the disease-related variations downstream of the genome and proteome. In this study, the serum metabolomics profile of PsA was compared with healthy controls, by nuclear magnetic resonance spectroscopy (^1^H-NMR). Twenty-nine PsA patients according to CASPAR criteria and DAPSA > 14 score plus 33 healthy controls matched for mean age and gender ratio, were enrolled. The sera metabolomics profile was analysed with a Varian Unity Inova 500 MHz NMR spectrometer, combined with multivariate statistical analysis (MVA). The MetaboAnalyst program was used to generate ROC curves and to perform the enrichment analysis. The OPLS-DA models exhibited a clear separation between the two groups, and the metabolomic profile of PsA patients was characterized by a significant increase in glucose and glycyl proline, and a significant decrease in alanine, glutamine, methionine, serine and the branched-chain amino acids leucine, isoleucine and valine. The ROC curve was 0.842 (95% CI 0.735–0.949), and a significant direct correlation of alanine and leucine levels with the DAPSA score was recorded. Finally, the enrichment analysis unveiled that in PsA patients, the most enriched pathways were glucose-alanine cycle, glycine and serine, glutathione, selenoamino acid, alanine and tryptophan metabolism, with a close relationship between pathways. The metabolomic profiling in PsA may offer additional insight to elucidate the metabolite unbalance on inflammatory burden, oxidative stress, energy and collagen metabolism, but also on peculiar features of the disease, such as metabolic disorders and diabetes.

**Supplementary Information:**

The online version contains supplementary material available at 10.1038/s41598-025-34101-4.

## Introduction

Psoriatic arthritis (PsA) is a chronic inflammatory disease characterized by a wide clinical heterogeneity, resulting from the variable involvement of diverse domains, including skin and nail psoriasis (PsO), arthritis, enthesitis, dactylitis, spondylitis and sacroiliitis^1^. Moreover, non-musculoskeletal and non-cutaneous manifestations may be associated with PsO and PsA, such as uveitis, inflammatory bowel disease, and other comorbidities, including obesity and metabolic syndrome, type 2 diabetes mellitus and cardiovascular disease^2^. Psoriatic arthritis (PsA) affects women and men equally, often leading to structural damage and reduced quality of life. It is a potentially disabling disease because delayed or insufficient treatment can cause lasting physical damage and disability^3^. Several genetic, circulating, and tissue factors have been studied as players in managing different aspects of PsA, including diagnosis and assessment of disease activity, severity, and response to treatment^4–9^. However, none of them has been extensively validated and then translated into routine clinical practice. PsA, is driven by a specific set of factors that promote an aberrant immune response, ultimately leading to chronic inflammation and tissue damage. Pivotal studies have highlighted the contribution of genetic susceptibility, environmental triggers, and systemic inflammation to its pathogenesis, but the precise mechanisms are still under investigation^10–14^. Among these drivers, metabolic factors including obesity, insulin resistance, and dyslipidemia, play a key role by facilitating the sustained mobilization, trafficking, and homing of immune cells into target tissues^15,16^. However, as a complex and multifactorial condition, PsA pathogenesis remains only partially understood. In oncology, the emergence of proteomics and metabolomics has opened new possibilities for using combinations of biomolecules to distinguish between different diseases or their stages^17^, which is not possible with only genetic markers. In readily accessible body fluids such as serum, temporal changes in metabolite profiles and their abundances are associated with disease, reflecting underlying abnormal cellular processes. These biochemical alterations could provide a direct measure of disease onset or severity, for example. Low-molecular-weight metabolites hold great promise in precision medicine for improving the diagnosis and monitoring of PsA. As sensitive indicators of phenotypic changes, they reflect the biochemical activity of tissues and provide insights into responses to a variety of genetic and environmental stimuli^18,19^. Similarly, metabolomics may offer additional insights into the metabolic pathways underlying the pathogenesis of chronic, immune-mediated processes that are characteristic of PsA, potentially revealing novel drug targets^20^. Although metabolomics provides a powerful platform for identifying molecules and pathways involved in multifactorial disease such as PsA, the literature review revealed that few studies have explored the association between the metabolome and PsA pathogenesis, with various amino acids and lipids identified as potential, but not fully validated, biomarkers. Armstrong et al. reported increased serum glucuronic acid concentrations in PsA patients relative to healthy controls^21^. Furthermore, most studies are comparative investigations with other autoimmune pathologies such as rheumatoid arthritis (RA) or psoriasis^22–26^ and will be mentioned in the discussion section of this article. Current methods for evaluating PsA disease activity are not accurate enough for precision medicine, and only a few studies have correlated serum metabolites with measures of PsA disease activity. Moreover, the major limits are intrinsic to the clinical assessment because they vary between studies, and the results are often not validated on a large scale. Among these, it was shown that trimethylamine-N-oxide (TMAO) positively correlated with PsA skin and peripheral joint activity^27^, and pro-inflammatory eicosanoids PGE2, HXB3 and 6,15-dk, dh, PGF1a and anti-inflammatory eicosanoids 11-HEPE, 12-HEPE and 15-HEPE correlated with joint disease score in PsA patients^28^. Recently, Koussiouris and colleagues found that some lipids (lysophosphatidylcholine and sphingomyelin) and other metabolites predict high disease activity from both low and moderate^29^. Choksi et al., with a metabolomic-based approach combined with machine learning algorithms, have identified potential metabolites associated with skin disease activity in PsA patients^30^. This study aimed to compare the metabolomic profile of PsA patients versus healthy controls and correlate circulating metabolites with the DAPSA score of patients with high disease activity.

## Materials and methods

### Patients and controls

The present study was based on the comparative metabolomic profiling of two groups of subjects: 29 consecutive patients with active psoriatic arthritis (PsA) and 33 healthy controls (HC). The PsA patients, recruited from a monocentric cohort, were diagnosed according to the ClASsification for Psoriatic ARthritis (CASPAR) criteria^31^ and classified as active if they had a Disease Activity PsA (DAPSA) score of > 14^32^. The demographic and clinical features of the enrolled individuals, and the treatment regimen of the PsA group are reported in Table 1.

**Table 1:** Clinical and demographic parameters of recruited cohorts.

	PsA (*n* = 29)	HC (*n* = 33)
Males, n (%)	14 (35.9%)	17 (40.5%)
Age at enrollment, mean (SD) years	53.2 (14.6)	53.1 (9.7)
Age at the PsA onset, mean (SD) years	44.9 (14.5)	-
Disease duration, mean (SD) years	5.6 (5.4)	-
Peripheral involvement, n (%)	29 (100%)	-
Axial involvement	1 (2.6%)	-
BMI, mean (SD)	27,8 (5.3)	-
cs-DMARDs, n (%)	18 (46.2%)	-
TNF-alpha inhibitors, n (%)	2 (5.1%)	-
Other b-DMARDs, n (%)	7 (17.9%)	-
HAQ, mean (SD) score	1.5 (0.6)	-
ESR, mean (SD) mm/h	25.7 (17)	-
CRP mean (SD) mg/L	8.2 (10.9)	-
DAPSA, mean (SD) score	25.2 (10.4)	-
Metabolic comorbidities		
Mellitus diabetes type 2, n (%)	6 (15.4%)	-
Hypercholesterolemia, n (%)	5 (12.8%)	-
Hypertriglyceridemia, n (%)	3 (7.7%)	-
Arterial hypertension, n (%)	9 (23.1%)	-
Obesity, n (%)	14 (35.9%)	-
Metabolic syndrome, n (%)	6 (15.4%)	-
Hyperuricemia, n (%)	4 (10.3%)	-

PsA, patients with active psoriatic arthritis, HC, healthy controls. BMI, body mass index. cs-DMARDs, conventional synthetic disease modifying anti-rheumatic drugs. b-DMARDs, biologic DMARDs. HAQ, health assessment questionnaire. ESR, erythrocyte sedimentation rate. CRP, C-reactive protein. DAPSA, disease activity PsA score.

Concomitant treatment with glucocorticoids was not allowed. The healthy control group was matched for mean age (± 2 yrs) and gender ratio with the PsA group. The study was conducted following the Good Clinical Practice standards and the Helsinki Declaration, and approved by the Ethical Committee of the University of Cagliari (protocol PG/2018/16313). Informed consent was obtained from all subjects involved in the study.

### Sample collection and metabolite extraction

Blood samples of PsA and HC were collected in tubes with a clot activator and placed vertically for 30 min at room temperature. The tubes were centrifuged at 2000 g for 15 min, and about 800 µL stored at − 80 °C until metabolomics analysis. The extraction of water-soluble metabolites from serum samples was performed based on the Folch, Lees, and Sloane-Stanley procedure^33^ and has been already described in previously published papers^34^. 400 µL of serum samples were resuspended in a chloroform/methanol mixture (1:1, v/v) (1,2 mL) and 175 µL of H_2_O. After centrifugation at 4500 rpm and 4 °C for 30 min, 1 mL of the hydrophilic phase was separated from the lipophilic one. The hydrophilic fraction enriched with low molecular weight water-soluble compounds was dried using a vacuum concentrator (Eppendorf, Hamburg, Germany) and subsequently stored at − 80 °C. Dried hydrophilic serum extracts were reconstituted in potassium phosphate buffer in D_2_O (690 µL, 100 mM, pH 7.4) and TSP (10 µL, sodium 3-trimethylsilyl-propionate-2,2,3,3,-d4) as chemical shift reference (δ 0.0) (98 atom % D, Sigma-Aldrich, Milan). An aliquot of 650 µL was subjected to ^1^H-NMR.

### ^1^H-NMR analysis

^1^H-NMR analysis of serum samples was conducted using a Varian UNITY INOVA 500 spectrometer operating at 499.839 MHz for proton and equipped with a 5 mm double resonance probe (Agilent Technologies, CA, USA). The spectra were acquired at 300 K with a spectral width of 6000 Hz, a 90° pulse, an acquisition time of 2s, and a relaxation delay of 3s. For each sample, 256 free induction decays were acquired into 64 K data points. The residual water signal was suppressed by applying a presaturation technique with low-power radiofrequency irradiation for 2s. Following Fourier transformation with 0.3 Hz line broadening and a zero-filling to 64 K, ACD Lab Processor Academic Edition (Advanced Chemistry Development, 12.01, 2010) was used to perform phase adjustment and baseline correction of ^1^H-NMR spectra as well as to set spectral chemical shift referencing on the TSP CH_3_ signal at 0.00 ppm.

### ^1^H-NMR data preprocessing and multivariate statistical analysis

Spectral integration between 0.80 and 8.50 ppm was carried out using the ACD Labs intelligent bucketing method^34^. A 0.04 ppm bucket width was defined with an allowed 50% looseness, resulting in buckets that ranged between 0.02 and 0.06 ppm in width. The degree of looseness allows the bucket width to vary over a particular value from the set bucket value. The intelligent bucket method contains an algorithm that identifies local minima in the spectra and sets the buckets accordingly. In this manner, a peak is integrated into one bucket, although it may be differently shifted in the spectra because of the pH effect, for instance. To eliminate the effect of variations in the presaturation of the residual water resonance, the spectral region between 4.70 and 5.20 ppm was excluded from the analysis. To reduce the effects of variable concentration among different samples, the spectral data set was normalized to the total area and subsequently imported into the SIMCA software (Version 17.0, Sartorius Stedim Biotech, Umea, Sweden). Furthermore, Pareto scaling method was applied to the variables (spectral data). More in detail, dividing each variable by the square root of the standard deviation, assigns greater weight to the NMR data variables with lower values, while it is not as extreme as using unscaled raw data^35^. To explore the NMR data, a multivariate statistical approach was used. Orthogonal partial least squares discriminant analysis (OPLS-DA) was employed to reduce model complexity and to highlight samples discrimination. OPLS-DA is a supervised classification technique and maximizes the covariance between the measured data of the peak intensities in NMR spectra (the X-variable) and the response of the class assignment (Y-variable) within the groups. A 7-fold cross-validation and “permutation test” (500 times) were used to evaluate the goodness of fit of the model. The permutation test was estimated by randomizing the Y-matrix (class assignment or continuous variables) while the X-matrix (peak intensity in NMR spectra) was fixed. The permutation plot then displays the correlation coefficient between the original y-variable and the permuted y-variable on the x-axis versus the cumulative R2 and Q2 on the y-axis and draws the regression line. The intercept is a measure of the overfit, Q2Y intercept value less than 0.05 indicates a valid model. The estimated predictive power of the models was indicated by R2Y and Q2Y values, which represent the fraction of the variation of Y-variable and the predicted fraction of the variation of Y-variable, respectively. Values of Q2 > 0.5 suggested a good prediction model. Metabolites with a Variables Important in the Projection (VIP) value greater than 1 were considered for evaluation of their role in class separation.

### Univariate statistical analysis for ^1^H-NMR data

The metabolite concentrations were quantified by using Chenomx NMR suite 7.1 (Chenomx Inc., Edmonton, Alberta, Canada). The statistical significance of the differences in metabolite concentrations was evaluated by using the Mann-Whitney U test and a p-value < 0.05 was considered statistically significant. Subsequently, to acquire the level of significance for multiple testing, the Benjamini-Hochberg adjustment was applied to the p-values^36^. Chenomx NMR Suite is an integrated software for identifying and quantifying metabolites in NMR spectra by using reference libraries^37^.

Receiver operator characteristic curves (ROC), sensitivity, specificity, and Area Under the ROC Curve (AUC) were obtained by MetaboAnalyst program *(*https://www.metaboanalyst.ca/*).* The Logistic Regression method was used to build the ROC curve using the “Tester” application. The accuracy of a test for correctly distinguishing PsA and HC patients from controls was indicated by AUC and 95% CI. To circumvent potential over-fitting when generating ROC curves from the combined model, a 100-fold cross-validation and permutation test were applied. Finally, enrichment and network analysis were performed with MetaboAnalyst V5.0 program.

## Results

^1^H-NMR spectroscopy coupled with multivariate data analysis was applied to investigate the metabolomic profile of serum samples for both PsA and healthy controls (HC). Metabolites were identified based on literature information and by using a dedicated library, such as the Human Metabolome Database (HMDB, http://www.hmdb.ca) and the 500 MHz library from Chenomx NMR suite 7.1. The whole ^1^H-NMR dataset was subsequently subjected to multivariate statistical analysis.

### Metabolomic comparison between PsA and healthy conditions

**Fig. 1:**
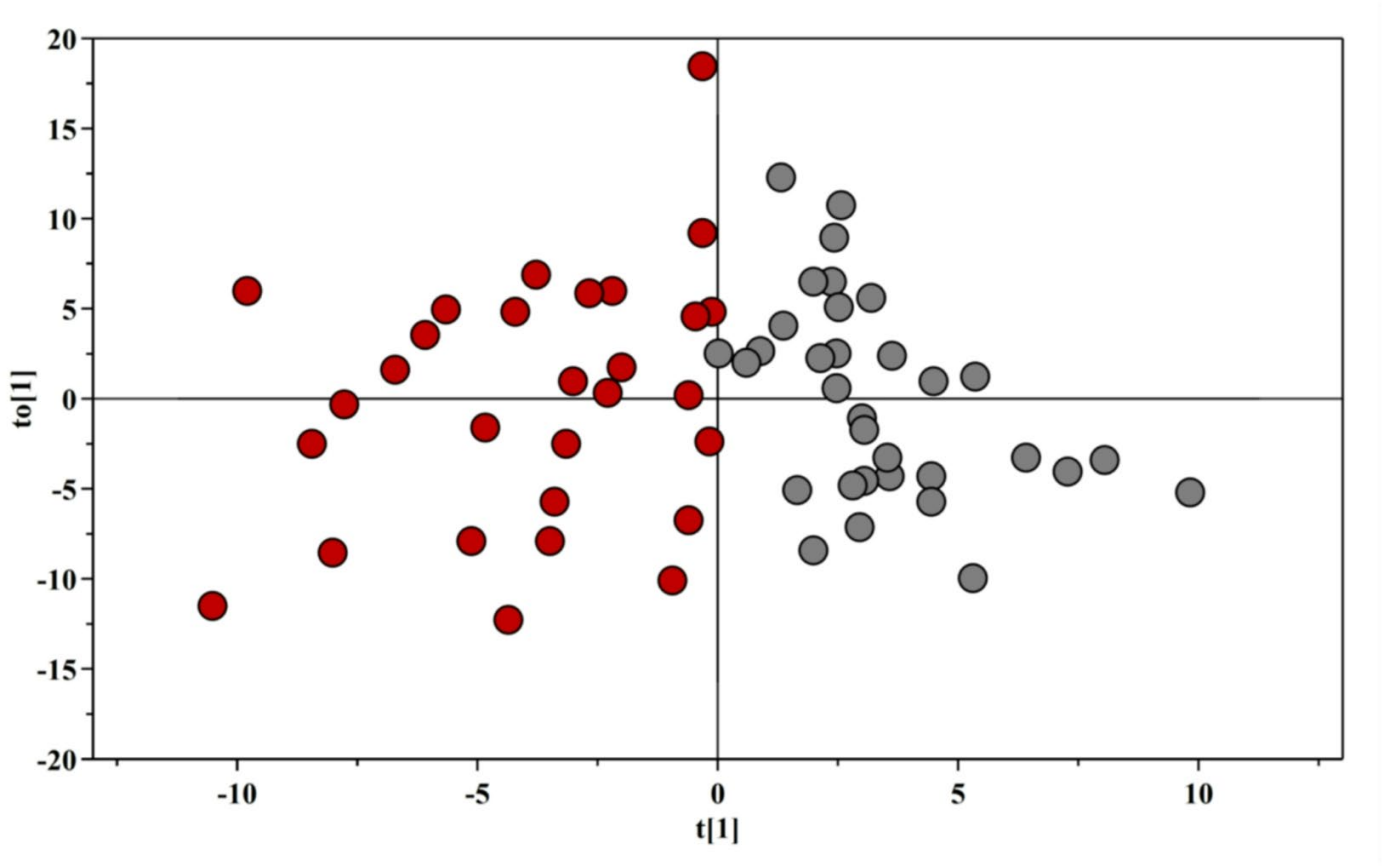
OPLS-DA scores plot of ^1^H-NMR spectra of serum samples. The red circles represent PsA subjects, while the grey circles represent HC subjects.

A supervised OPLS-DA was conducted to evaluate differences in the serum metabolomic profile between PsA patients and the HC group. OPLS-DA scores plot (Fig. 1) showed a great separation between PsA and HC, indicating differences in the metabolomics profile between the two groups.

The OPLS-DA model was established with one predictive and one orthogonal components and showed good values of R2X, R2Y, and Q2 (Table 2). The validity of the OPLS-DA model was performed with a permutation test (500 times) (Figure S1). The test results are reported in Table 2 and indicate the statistical validity of the OPLS-DA model.

**Table 2:** Summary of the statistical parameters of OPLS-DA models.

Groups	OPLS-DA models				Permutation (500 times)*	
	Components^a^	R2Xcum^b^	R2Ycum^c^	Q2cum^d^	R2 intercept	Q2 intercept
PsA *versus* HC	1P + 1O	0.449	0.664	0.577	0.253	-0.329
PsA: cs-DMARDs *versus* other pharmacological treatments	1P + 1O	0.319	0.208	0.011	0.287	-0.233

^a^The number of Predictive and Orthogonal components used to create the OPLS-DA statistical models. ^b, c^R2X and R2Y indicated the cumulative explained fraction of the variation of the X block and Y block for the extracted components. ^d^Q_2_ cum values indicated the cumulative predicted fraction of the variation of the Y block for the extracted components. ^*^A Q2 intercept value less than 0.05 is indicative of a valid model.

**Fig. 2:**
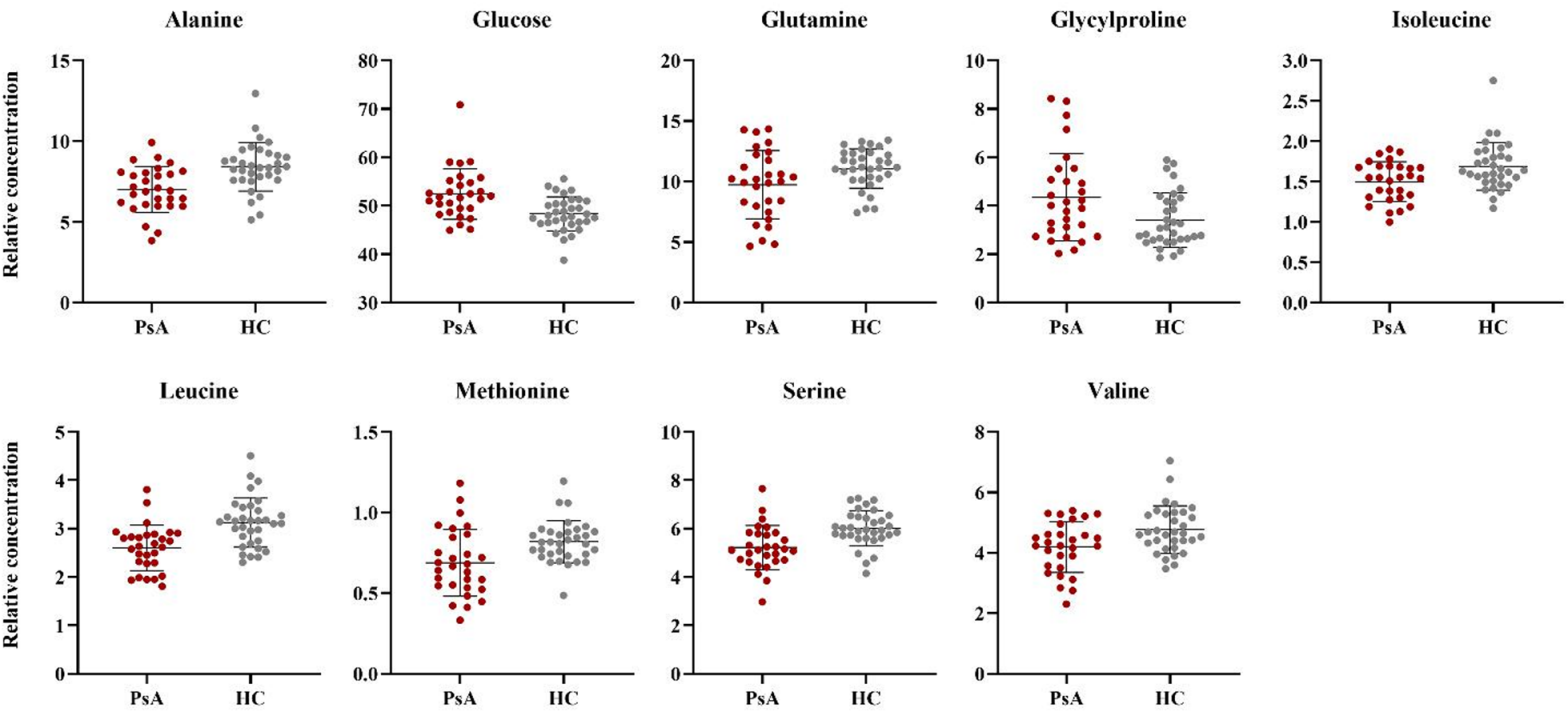
Scatter plots showing the metabolites concentration of PsA patients compared to HC. All plotted metabolites are characterized by VIP > 1 and a p-value < 0.05 (with Benjamin-Hochberg correction) In detail: alanine p-value = 0.016; glucose p-value = 0.022; glutamine p-value = 0.044; glycylproline p-value = 0.050; isoleucine p-value = 0.033; leucine p-value = < 0.001; methionine p-value = 0.027; serine p-value = 0.011; valine p-value = 0.038.

The most informative metabolites in distinguishing between the two groups are identified by the Variable Importance in the Projection score higher than 1 (VIP > 1) and are reported in Table 3. For each metabolite, the relative concentration in the PsA patients and HC group was calculated and subjected to univariate statistical analysis. The relative concentrations of significantly altered metabolites (p-value < 0.05) were compared using scatter plots (Fig. 2).

**Table 3:** Metabolites altered in active PsA vs healthy condition.

Metabolites [mM]^a^	Mean PsA ± SD	Mean HC ± SD	*p*-value	*p*-value correct*
2-aminobutyrate	1.911 ± 0.314	1.900 ± 0.273	0.768	–
Alanine	7.010 ± 1.416	8.402 ± 1.499	**< 0.001**	0.016
Anserine	2.509 ± 0.739	2.753 ± 0.635	0.147	–
Glucose	52.43 ± 5.242	48.340 ± 3.514	**< 0.001**	0.022
Glutamine	9.739 ± 2.817	11.080 ± 1.618	**0.036**	0.044
Glycilproline	4.353 ± 1.801	3.411 ± 1.127	**0.037**	0.05
Isoleucine	1.500 ± 0.245	1.688 ± 0.294	**0.016**	0.033
Leucine	2.601 ± 0.470	3.124 ± 0.508	**< 0.001**	< 0.001
Methionine	0.6879 ± 0.207	0.8190 ± 0.130	**0.002**	0.027
Serine	5.2170 ± 0.914	6.008 ± 0.729	**< 0.001**	0.011
Threonate	6.601 ± 1.109	6.412 ± 0.997	0.339	–
Tryptophan	1.252 ± 0.239	1.349 ± 0.217	0.087	–
Valine	4.189 ± 0.837	4.769 ± 0.781	**0.016**	0.038

^a^For each sample, the relative concentration was obtained by normalizing the molar concentration of each metabolite to the total molar concentration of all metabolites. The metabolites characterized by VIP > 1 (Variable Importance on Projection), potentially responsible for the separation of PsA patients from HC are evaluated using a Mann–Whitney U test corrected by Benjamini-Hochberg. p-value for significance is < 0.05 (bold); *p-value with Benjamini-Hochberg correction.

As shown in Table 3 and Fig. 2, the metabolomic profile is characterized by a significant increase in glucose and glycylproline in PsA patients compared to HC. Furthermore, a significant decrease in alanine, glutamine, methionine, serine and the branched-chain amino acids (BCAAs: isoleucine, leucine, and valine) was observed in PsA. An additional OPLS-DA analysis was conducted to evaluate the potential effect of different pharmacological treatments on the metabolomic profile of PsA patients. The OPLS-DA model was built with a predictive and an orthogonal component (1P + 1O) and showed R2Xcum, R2Ycum, and Q2cum values of 0.319, 0.208 and 0.011, respectively (Table 2). Statistical parameters reveal a low predictive power, suggesting that the type of treatment does not substantially impact on the metabolic profile. These findings indicate that disease status is likely the primary determinant of metabolic alterations.

**Fig. 3:**
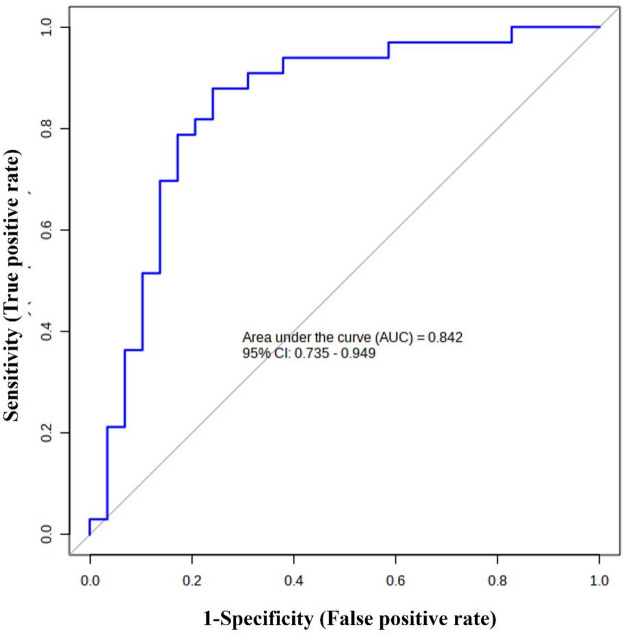
Receiver operating characteristic (ROC) analysis. Area under the curve (AUC) was found to be 0.842. *CI* confidence interval.

A ROC curve was built using only the metabolites with a significant statistical variation, and the area under the curve of the ROC analysis was found to be 0.842 (95% CI: 0.735–0.949), indicating high predictive accuracy of the model and thus a good discriminative ability of the metabolites to accurately differentiate between subjects with PsA and HC (Fig. 3). Table S1a shows the summary of each metabolite used to obtain the model, and Table S1b shows the performance values of the logistic regression models. The predictive algorithm developed using the abovementioned nine significant metabolites was as follows: Logit (P) = ln (P/ (1-P)) = – 61.645 + 2.991 Leucine + 2.08 Serine + 1.735 Alanine + 0.394 Glucose + 6.134 Methionine – 0.313 Isoleucine – 0.072 Valine + 0.256 Glycylproline + 0.346 Glutamine. P is Pr (y = 1/x). The best threshold (or cutoff) for predicted P was 0.38.

### Altered metabolites and DAPSA score

**Fig. 4:**
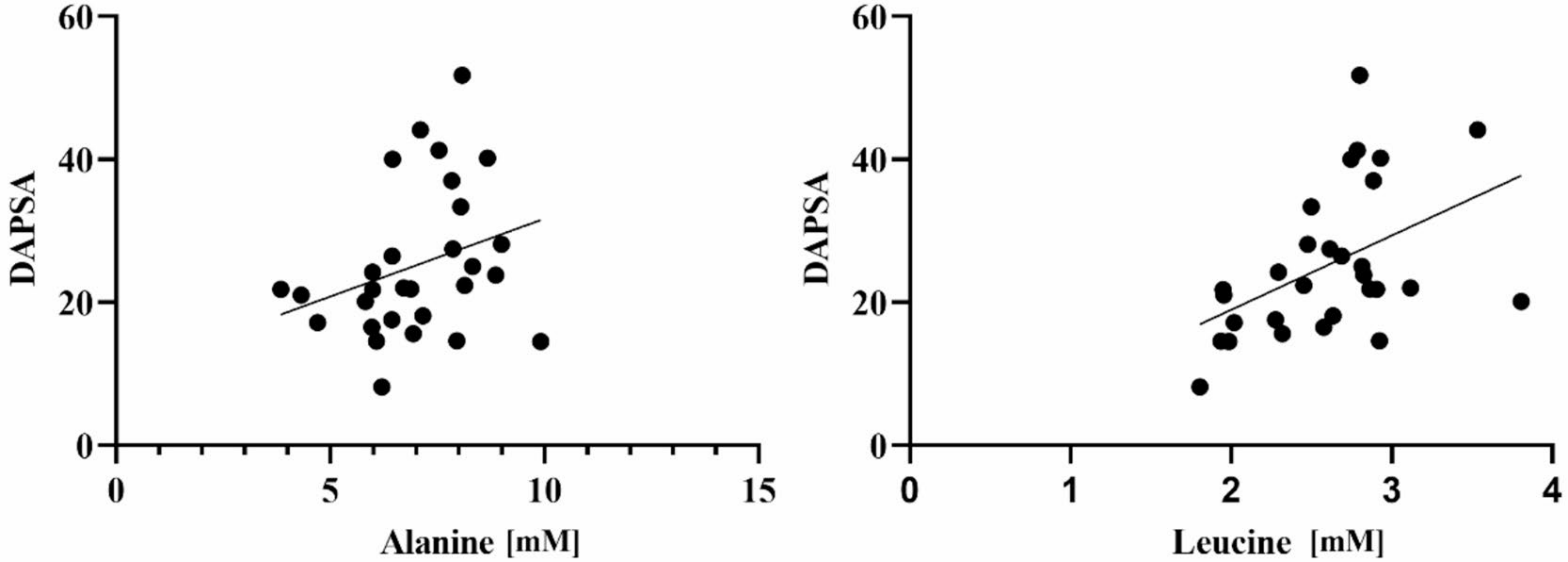
Spearman correlation of DAPSA score with the concentration of metabolites having p-value < 0.05. Two metabolites showed a correlation with disease activity: alanine (*p* 0.039) and leucine (*p* 0.007).

The Spearman correlation was applied to statistically significant metabolites and the DAPSA score of PsA. In Fig. 4, correlation plots for the metabolites and DAPSA score having a p-value of < 0.05 are shown, while Table 4 indicates which correlation tests reached statistical significance. A linear positive correlation was observed with alanine and leucine concentration and DAPSA score for PsA (Fig. 4 and Table 4).

**Table 4:** Significant metabolites and spearman correlation with DAPSA score.

Metabolites	DAPSA score		
	*r*	95% confidence interval	*p*-value
Alanine	0.384	0.009 to 0.664	**0.039**
Glucose	-0.282	-0.595 to 0.105	0.137
Glutamine	0.137	-0.251 to 0.488	0.477
Glycylproline	-0.154	-0.501 to 0.235	0.424
Isoleucine	0.350	-0.030 to 0.641	0.062
Leucine	0.487	0.136 to 0.730	**0.007**
Methionine	0.111	-0.276 to 0.468	0.563
Serine	0.0541	-0.328 to 0.421	0.780
Valine	0.321	-0.062 to 0.622	0.089

The metabolites characterized by VIP > 1 (Variable Influence on Projection) and p-value < 0.05 (Table 3), potentially responsible for the separation of PsA patients from HC are evaluated for Spearman correlation (“r”) with DAPSA score. p-value for significance is < 0.05 (bold).

### Enriched pathways and connected networks

**Fig. 5:**
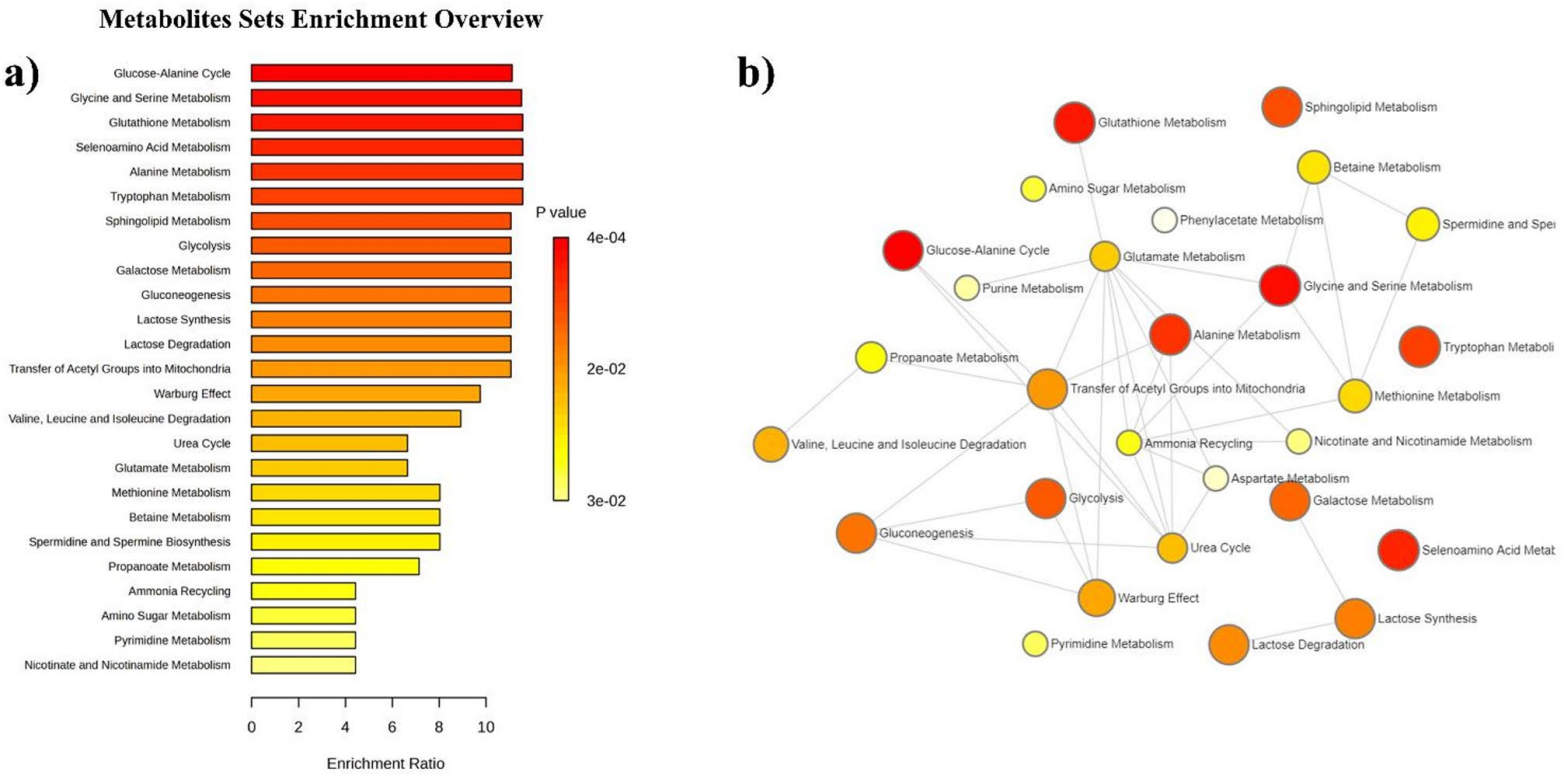
Enrichment analysis: (**a**) list of the most significantly discriminant pathways between PsA and HC; (**b**) network analysis illustrating the most significant relationships between perturbed biochemical pathways in PsA. The size and the color of each metabolic pathway reflect the pathway enrichment ratio and p-value, respectively.

Finally, the enrichment analysis unveiled that the top five enriched pathways are glucose-alanine cycle, glycine, and serine, glutathione, selenoaminoacid, alanine and tryptophan metabolism, as reported in Fig. 5a. The network view showed the most significant relationships between perturbed biochemical pathways in PsA, for example, between glutathione, alanine metabolism, and glycine and serine metabolism via glutamate metabolism, as well as between glucose-alanine cycle and alanine metabolism via the transfer of acetyl groups into mitochondria (Fig. 5b).

## Discussion

Metabolomics is a powerful technique that could help to increase understanding of molecular pathways and pathogenesis underlying multifactorial disease such as PsA, that results from the interaction between diverse biological and environmental factors. This study offers original and valuable data on the role of metabolomics as a key source of insights into the pathological mechanisms underlying PsA and its heterogeneous phenotype, allowing for a better understanding of the disease-related variations downstream of the genome and proteome.

First, this study demonstrated a great separation in the metabolomics profile between the PsA and the HC, highlighting altered metabolites and concerning biochemical pathways. In particular, a significant increase in glucose and glycyl proline, and a significant decrease in alanine, glutamine, methionine, serine and BCAAs were found in the PsA when compared with the HC.

The increased concentration of glucose observed in this study was already described and may be linked with the well-known association of PsA with type 2 diabetes mellitus (DT2), recorded with a higher prevalence (from 6.1 to 20.2%) of DT2 compared with the general population^38^. This unbalance is in keeping with our PsA cohort clinical features, in which the most recorded metabolic comorbidities (see Table 1) were obesity (35.9%), hypertension (23.1%), hypercholesterolemia (12.8%), DT2 (15.4%) and metabolic syndrome (15.4%). Metabolic disorders in PsA arise from various pathomechanisms, and primarily an inflammation of both skin, joints and enthesis seems to influence glucose metabolism. Inflammation modulates insulin sensitivity and disrupts insulin signaling pathways^39^.

Glycylproline is a dipeptide composed of glycine and proline, and is an end product of collagen metabolism. Elevated levels of circulating glycylproline, often linked to reduced prolidase activity, may indicate increased collagen degradation or turnover, which is associated with disorders affecting connective tissues, joint health, or skin integrity^40,41^.

To our knowledge, this is the first demonstration of increased levels of this metabolite in PsA: glycylproline monitoring could provide insights into the body’s collagen metabolism and overall tissue health.

Glutamine, a non-essential amino acid derived from glutamic acid, represents the most abundant free amino acid in human blood and it is an immunomodulator, affecting T-cell-mediated immune response, and might be depleted by immune cells due to the heightened demands for protein biosynthesis^42^. The proliferation, differentiation, and effector functions of lymphocytes, macrophages, and neutrophils are highly dependent on glutamine availability^43–45^. Decreased glutamine levels were previously proposed as indicative of PsA to discriminate from Rheumatoid Arthritis (RA)^46^.

Madsen et al. highlighted reduced glutamine levels between PsO patients and healthy controls^21^. Furthermore, glutamine concentrations were negatively correlated with the Psoriasis Area and Severity Index (PASI) score^45^. This association may be explained by the increased expression of keratinocyte transglutaminase in psoriatic lesions^47^, and/or by the high rate of keratinocyte hyperproliferation characteristic of psoriasis that elevates the cellular demand for amino acids^48^. These considerations and the glutamine decrease reported in this study can support the hypothesis that glutamine monitoring and supplementation could be beneficial for PsA patients, at least for patients comorbid with metabolic diseases.

Branched-chain amino acids (BCAAs), valine, leucine, and isoleucine are essential amino acids in both animals and humans, known for their remarkable metabolic and regulatory roles. Alterations in BCAA metabolism have been extensively studied in various pathological conditions^49^. It is interesting that BCAAs are metabolic regulators not only in protein synthesis but also in lipid and glucose metabolism and may promote diabetes via hyperaminoacidaemia^50^, since PsA is known to be associated with an elevated risk of diabetes when accounting for obesity and lifestyle factors^51^. However, some other studies present conflicting results^52,53^, and no affordable data regarding BCAAs unbalance in PsA are available.

The connection between BCAAs and arthritis is complex, and other factors like inflammation, oxidative stress, and metabolic pathways play a significant role in the development of this condition. We suppose that our BCAAs measurement are in line with rheumatoid arthritis, in which the levels of valine, leucine, and isoleucine have been reported as decreased in the patient’s sera^54,55^. Additionally, in a rat model of adjuvant-induced arthritis, valine and leucine showed lower concentrations in plasma that might result from its consumption as an alternative substrate donor of the TCA cycle, to support the heightened energy demand^56,57^.

About the association between PsA and metabolic disorders, it is needed to first consider the percentage of diabetic patients in our cohort (15.4%), and speculate about the reduced BCAAs viability because of its high consumption. In our opinion, the well-note PsA comorbidities could have a minor impact on BCAAs unbalance (not all patients develop metabolic syndrome), with a greater effect of arthritis as the primary symptom of our cohort: we can suppose that reported results are inflammation-driven, and the activated catabolism associated with this condition, contributes to the decreased BCAAs circulating levels, maybe because an higher uptake of these amino acids to fuel the immune response and cellular metabolism.

When assessing the relationship between metabolite levels and high PsA disease activity, a significant direct correlation was found between alanine and leucine levels and DAPSA score, which is a validated composite outcome measure based on the number of swollen and tender joints, CRP levels, and the patient’s assessment of the pain and activity related to the disorder^58^. A serum metabolomic profiling in Rheumatoid Arthritis by Narasimhan et al., demonstrates that alanine levels were associated with synovial B-lymphocyte stimulator expression and leucine with synovial expression of IL-1β and IL-8^59^.

Research suggests that alanine may be linked to the development of Osteoarthritis, a degenerative joint disease, particularly in the knee and hip joints^60,61^. About amino acid leucine, some research findings in Rheumatoid Arthritis demonstrate a possible role of the Leucine-Rich Alpha-2 Glycoprotein (LRG) as biomarker for assessing disease activity^60–62^. Additionally, LRG is upregulated in severe knee Osteoarthritis and may be a key player in joint inflammation and fibrosis in this condition^63^.

To our knowledge, this is the first description in PsA disease of these metabolites correlating with the high inflammatory burden: in that milieu, circulating free amino acids may reflect disease-associated protein catabolism.

Although in the comparison PsA vs. HC, the alanine and leucine values are globally down (Fig. 2), when the intra-condition evaluation is made (Fig. 4), the variation with an inverse trend is recorded. We cannot provide the biological significance to reconcile the apparent incongruence between the low-metabolite’s association with PsA as disease and its positive correlation with the activity score (e.g. compensatory mechanism because of the high consumption?): further studies are needed to validate the biological role and availability of these molecules on PsA disease and its severity^64,65^.

The most enriched pathways were glucose-alanine cycle, glycine and serine, glutathione, selenoaminoacid, alanine and tryptophan metabolism. Furthermore, the network analysis showed a close relationship between various metabolic pathways, for example, between glutathione and alanine metabolism via glutamate metabolism, as well as between the metabolism of glycine and serine and alanine metabolism via glutamate metabolism and that of glucose-alanine cycle and alanine metabolism via transfer of acetyl groups into mitochondria.

In this study, the most discriminant pathways between PsA patients and healthy controls showed a close relationship and play a critical role in various biological processes. The imbalance of several amino acids and metabolites discovered in our study is involved in oxidative stress and energy metabolism, tissue health, but is also pivotal for immune cell activation.

In detail, the glucose-alanine cycle plays a key role in energy metabolism during muscle activity. In this process, muscle protein is degraded, generating more glucose for ATP production and supplying muscle contraction. It allows pyruvate and glutamate to be transported out of muscle tissue to the liver where gluconeogenesis takes place to supply the muscle tissue with more glucose (here reported as increased in PsA vs. HC), as reported in the database (National Center for Biotechnology Information (2025). PubChem Pathway Summary for Pathway SMP0087221, Glucose-Alanine Cycle, Source: PathBank. Retrieved March 19, 2025 from https://pubchem.ncbi.nlm.nih.gov/pathway/PathBank:SMP0087221).

Furthermore, recent findings in PsO have highlighted serine/glycine as a metabolic hub controlling both keratinocyte proliferation and inflammatory cell expansion^66^, which is in line with the skin damage involvement also in our PsA patients (no patients *sine* psoriasis were enrolled in this study).

Glutathione (γ-glutamyl-cysteinyl-glycine; GSH) is the most abundant low-molecular-weight thiol, and GSH/glutathione disulfide is the major redox couple in animal cells. Glutathione plays important roles in antioxidant defense, nutrient metabolism, and regulation of cellular events including gene expression, DNA and protein synthesis, cell proliferation and apoptosis, signal transduction, cytokine production and immune response^67^. For this purpose, glutathione (GSH) plays a pivotal role in the activation of T lymphocytes and polymorphonuclear leukocytes, thereby contributing critically to the initiation and regulation of effective immune responses^68^. Its deficiency contributes to oxidative stress, which plays a key role in aging and the pathogenesis of many diseases, including the PsA^69,70^.

Organic selenium (Se)-containing amino acids, such as selenocysteine and selenomethionine, can provide antioxidant benefits by acting both as direct antioxidants as well as a source of selenium for the synthesis of selenium-dependent antioxidants and repair proteins (e.g., glutathione peroxidases, thioredoxin reductases, methionine sulfoxide reductases)^71^. A direct comparison to prior studies is difficult, since the database on selenium status in rheumatic diseases is small, and a comparison of protein biomarkers of selenium status in the different patient groups has not been reported before. This knowledge gap concerns especially PsA. The recently published analytical study of Whal et al. determined the selenium status by assessing three selenium biomarkers in the serum of patients with a diagnosis of Juvenile Idiopathic Arthritis, PsA, or RA^72^. A comparison of the results to a reference cohort of European healthy subjects indicates selenium deficiency in most patients, which is likely to be relevant to the severity and progression of the disease. The finding of disrupted selenium metabolism in patients suggests a potential feed-forward cycle of pharmacological treatment, oxidative stress, renal damage and impaired selenoprotein expression. The markedly reduced selenoprotein status observed in inflammatory rheumatic musculoskeletal diseases supports the rationale for controlled interventional studies using selenium supplementation, particularly in patients with confirmed selenium deficiency.

Finally, there is an increasing interest in the pathophysiological role of the kynurenine pathway of tryptophan metabolism in the regulation of immune function and inflammation^73^. Tryptophan (Trp), an essential amino acid, functions not only as a fundamental substrate for protein biosynthesis but also as a precursor in several critical metabolic pathways and molecules such as serotonin. Among these, the kynurenine pathway is notably implicated in modulating immune responses and has been increasingly associated with chronic inflammatory conditions^74^. In RA, alterations in the Trp metabolism contribute actively to the pathogenesis and could be considered as a new therapeutic avenue^75,76^. Again, given the emerging role of tryptophan and its metabolites in immune function, Trp conversion into indole-3-acetaldehyde and then indole-3-acetate, represents a mechanism by which the gut microbiome potentially influences the development of axSpA^77^. Additionally, the serotonin pathway of Trp metabolism plays a crucial role in the regulation of mood and mental health, especially in depression and anxiety^78,79^. For this purpose, it could be noteworthy to functionally validate its possible role on PsA depression and anxiety as a described comorbidity^80^ considering also that the tryptophan-kynurenine metabolism has been proposed as a gut-brain link for depression in IBD, itself comorbidity of PsA^81^. Further studies need to be designed for this purpose. Despite the limitation of this study due to the restricted metabolic coverage of only hydrophilic molecules, our findings suggest that the molecule metabolic differences in PsA sera compared to healthy conditions contribute to elucidating the role of metabolite unbalance on the inflammatory burden, oxidative stress, energy and collagen metabolism but also on peculiar features of PsA disease, as metabolic disorder and diabetes. Although the panel of metabolites identified in this manuscript is of scientific interest and warrants further investigation, their biological roles have not been validated here. Additional studies are required to biologically confirm their involvement in PsA.

## Conclusions

The functional effects of metabolites depend on their abundance in the cell subtype and tissue microenvironment that produces and expands them. A metabolite can be increased because of its excessive production and accumulation, while decreased levels may be the result of its constant consumption and/or insufficient making.

A closer examination of circulating metabolites contributes to our understanding of the pathophysiology and may potentially provide markers of PsA disease: integrating this knowledge with current precision medicine efforts, clinicians could be guided in developing personalized interventions (for example, with aminoacidic monitoring and/or supplementation), based on the patient’s metabolic profile. However, further studies involving a larger patient cohort and metabolite’s biologic validation are necessary to enable the implementation of an external test set, which is essential to assess the model’s reliability.

## Supplementary Information

Below is the link to the electronic supplementary material.

Supplementary Material 1

## Data Availability

The dataset will be available on reasonable request to the corresponding author.

## Abbreviations

PsA: Psoriatic arthritis
PsO: Psoriasis
RA: Rheumatoid arthritis
HC: Healthy control
CASPAR: ClASsification for psoriatic ARthritis
DAPSA: Disease activity PsA
PCA: Principal component analysis
OPLS-DA: Orthogonal partial least squares discriminant analysis
VIP: Variable influence on projection
ROC: Receiver operator characteristic curves
AUC: Area under the curve
DT2: Diabetes type 2
BCAAs: Branched-chain amino acids
CRP: C-reactive protein
GSH: Glutathione
Trp: Tryptophan
