# Supplementary material for: The metabolomic profile of psoriatic arthritis patients unveils the unbalance of disease-related molecules and pathways
Source: Sci Rep. 2025 Dec 30;16:3985. doi: 10.1038/s41598-025-34101-4 (PMC12855187; doi:10.1038/s41598-025-34101-4)
Supplement: Supplementary file 1 — Supplementary Material 1 [file 41598_2025_34101_MOESM1_ESM.docx]

**The metabolomic profile of psoriatic arthritis patients unveils the unbalance of disease-related molecules and pathways**

M. M. Angioni^1^, C. Piras^2*^, V. P. Leoni^2^, A. Floris^1^, M. Spada^2^, K. Lilliu^2^, M. Congia^1^, E. Chessa^1^, M. Piga^1^, L. Atzori^2§^ and A. Cauli^1§^

^1^Rheumatology Unit, AOU and University of Cagliari, Department of Medical Sciences and Public Health, Monserrato, Cagliari, Italy

^2^Clinical Metabolomics Unit, University of Cagliari, Department of Biomedical Sciences, Monserrato, Cagliari, Italy

*****Correspondence: [cristina.piras@unica.it](mailto:cristina.piras@unica.it), University of Cagliari

§These authors contributed equally to this work


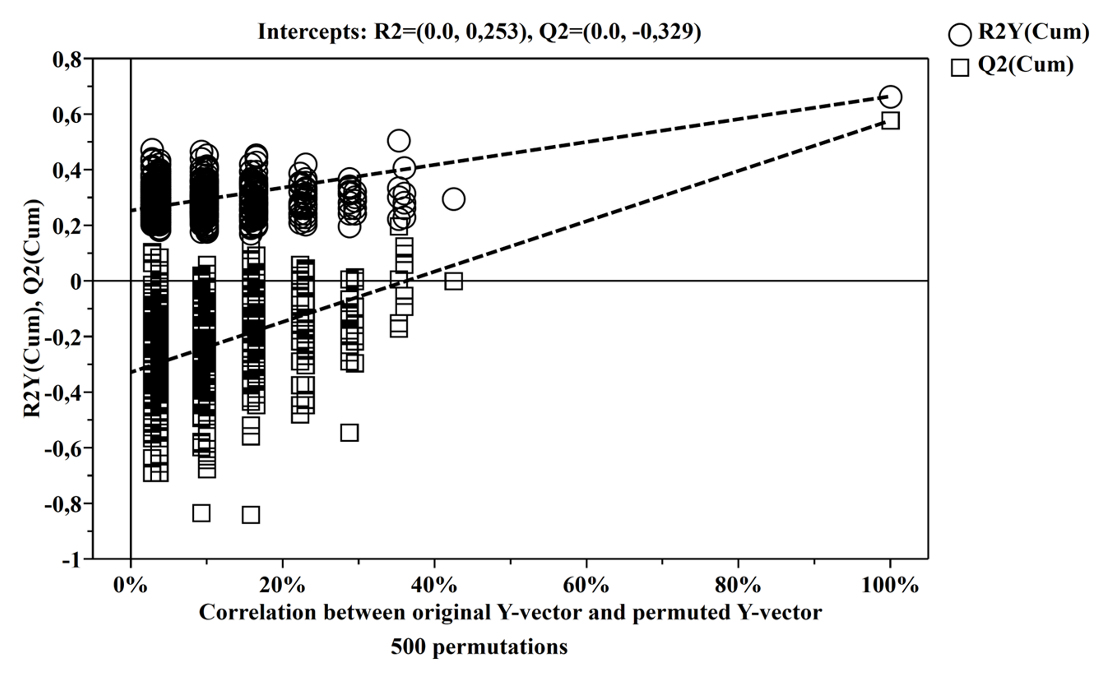


**Figure S1**. Validation plots of OPLS-DA model using a permutation test (n = 500). The horizontal axis shows the correlation between the permuted and actual data, while the vertical axis displays the cumulative values of R2 and Q2. The intercept gives an estimate of the overfitting phenomenon.

| **Table S1. Significantly altered metabolites used in the logistic regression model.**  a) Summary of each metabolite responsible for separation between PsA and HC. These metabolites were used to produce the linear regression model and b) performance values of the logistic regression models following 10-Fold Cross Validations | | | | | |
| --- | --- | --- | --- | --- | --- |
| **a)** | | | | | |
|  | **Estimate** | **Std. error** | **z value** | **Pr (>\|z\|)** | **Odds** |
| Intercept | -61.645 | 29.097 | -2.119 | 0.034 | - |
| Leucine | 2.991 | 1.657 | 1.805 | 0.071 | 19.9 |
| Serine | 2.08 | 0.717 | 2.901 | 0.004 | 8 |
| Alanine | 1.735 | 0.635 | 2.731 | 0.006 | 5.67 |
| Glucose | 0.394 | 0.297 | 1.324 | 0.185 | 1.48 |
| Methionine | 6.134 | 2.856 | 2.148 | 0.032 | 461.06 |
| Isoleucine | -0.313 | 2.577 | -0.121 | 0.903 | 0.73 |
| Valine | -0.072 | 0.883 | -0.081 | 0.935 | 0.93 |
| Glycylproline | 0.256 | 0.542 | 0.472 | 0.637 | 1.29 |
| Glutamine | 0.346 | 0.325 | 1.066 | 0.286 | 1.41 |
| **b)** |  |  | |  | |
|  | **AUC, 95% CI** | **Sensitivity** | | **Specificity** | |
| Training/Discovery | 0.935 (0.914 - 0.956) | 0.946 (0.920 - 0.972) | | 0.820 (0.773 - 0.867) | |
| 10-fold Cross-Validation | 0.842 (0.735 - 0.949) | 0.879 (0.879 - 0.990) | | 0.759 (0.603 - 0.914) | |
